# Insights into the Mechanism by Which Vacancy Defects Influence the Electrical and Piezoresistive Properties of Graphene

**DOI:** 10.3390/nano16070439

**Published:** 2026-04-03

**Authors:** Shuaituan Wang, Mengwei Li, Shengsheng Wei, Qiqi Dong, Guangjun Xing, Zhibin Wang, Junqiang Wang

**Affiliations:** 1 Shanxi Key Laboratory of Graphene Sensing Materials and Devices, North University of China, Taiyuan 030051, China; s202306094@st.nuc.edu.cn (S.W.); lmw@nuc.edu.cn (M.L.); s202306033@st.nuc.edu.cn (Q.D.); sz202306176@st.nuc.edu.cn (G.X.); 2 Academy for Advanced Interdisciplinary Research, North University of China, Taiyuan 030051, China; wangzhibin@nuc.edu.cn; 3 School of Instrument and Electronics, North University of China, Taiyuan 030051, China; 4 School of Semiconductors and Physics, North University of China, Taiyuan 030051, China

**Keywords:** graphene, vacancy defect, strain, fermi velocity, piezoresistive effect

## Abstract

Owing to its exceptional mechanical and electrical properties, graphene is regarded as an ideal sensing material for piezoresistive pressure sensors. However, vacancy defects inevitably introduced during graphene preparation and transfer significantly alter its electrical characteristics and piezoresistive performance. Based on first-principles calculations, this work systematically investigates the influence of mono-, di-, and tri-vacancy defects on the electrical and piezoresistive properties of graphene. The results indicate that di- and tri-vacancy defects reconstruct into 5-8-5 and 5-10-5 configurations during relaxation. Mono-, di-, and tri-vacancy defects effectively open bandgaps in graphene, yielding values of 0.62, 0.48, and 0.72 eV, respectively. The mono-vacancy introduces localized defect states near the Fermi level, the di-vacancy shifts the Dirac point from K to M, and the tri-vacancy moves it along the K-Γ path, eventually placing it between K and Γ. The application of strain not only widens the bandgap of defective graphene but also induces the movement of defect energy levels toward the band edges in the mono-vacancy system. All three defect types enhance the piezoresistive effect, with the tri-vacancy defect showing the most pronounced enhancement—boosting the gauge factor by a factor of 5.58. These findings provide a theoretical foundation for optimizing graphene-based pressure sensors.

## 1. Introduction

Due to their high sensitivity [1,2,3,4], miniaturization [5,6], low power consumption [7,8], and easy direct readout [9], piezoresistive pressure sensors have gained widespread interest in micro-electro-mechanical system (MEMS)-based smart sensing applications. Among various sensing materials developed for such sensors, graphene, a two-dimensional carbon nanomaterial, has been extensively employed owing to its exceptional mechanical and electrical properties [10,11,12,13]. Compared with silicon-based sensors, graphene sensors also exhibit superior temperature compatibility [14]. However, current studies still face several challenges. Specifically, defects such as vacancy defects [15,16,17], wrinkles [18,19], and dopant atoms [20,21], among others, are inevitably introduced during the growth and transfer processes of graphene [22]. Furthermore, the defect-induced modifications in the electrical and piezoresistive properties of graphene significantly affect the overall sensing performance, including sensitivity, linearity, and dynamic response. Therefore, an in-depth investigation into the electrical and piezoresistive properties of defective graphene is essential for optimizing the performance of graphene-based piezoresistive sensors.

In recent years, researchers have conducted extensive research on the electrical properties and piezoresistive effects of graphene containing defects. For instance, Wahid et al. employed low-temperature HFCVD to introduce high-density wrinkles and nanoparticle defects in graphene, showing that the resulting enhanced charge carrier scattering and modulated tunneling distances significantly improve sensor sensitivity to 0.0045 kPa^−1^ [23]. Haniff et al. utilized NH_3_/Ar plasma treatment to introduce nitrogen-doped defects into graphene, resulting in an order of magnitude enhancement in sensor sensitivity [24]. Mo et al. developed an MXene-doped graphene pressure sensor, which leverages the synergistic reduction in both carrier concentration and mobility to induce an increase in resistivity, thereby enhancing the sensitivity [25]. Esmaeil et al. have also conducted studies on properties of graphene such as carrier concentration and conductivity [26,27,28]. Although previous studies have elucidated the influence of wrinkles, dopants, and other defects on graphene-based pressure sensors from both experimental and theoretical perspectives, research on vacancy defects—a common and typical type of defect [29]—have largely been limited to their fundamental electrical properties. The piezoresistive effects of such vacancies and the underlying mechanisms by which they influence sensor performance have not yet been systematically investigated. Experimentally, several methods have been developed to introduce and control vacancy defects in graphene, such as plasma treatment [30], particle irradiation [31], and chemical etching [32]. These techniques can effectively modulate the density and distribution of defects, providing potential experimental routes for realizing periodic vacancy lattices and offering valuable guidance for theoretical studies.

In this study, first-principles calculations were employed to systematically investigate graphene containing mono-vacancy, di-vacancy, and tri-vacancy defects. Based on the analysis of material properties, we further revealed the intrinsic relationship between vacancy defect configuration, electronic band structure, Fermi velocity, and piezoresistive response. Structural relaxation shows that vacancy defects result in local lattice distortion and reconstruction, where di-vacancy and tri-vacancy defects generate 5-8-5 and 5-10-5 configurations. Combined calculations of the electron localization function (ELF), band structure, and orbital-projected density of states (PDOS) reveal the mechanism by which defects regulate electron localization behavior and the evolution and orbital contributions of electronic states. Through the strain-induced modulation of electronic band structure and Fermi velocity, the effects of different defect configurations on the piezoresistive response were quantitatively elucidated from a theoretical perspective, systematically revealing the piezoresistive characteristics of defective graphene and their underlying microscopic mechanisms. This study can supply critical theoretical guidance for the design of high-performance piezoresistive sensors.

## 2. Computational Conditions

### 2.1. Computational Methods

First-principles calculations based on density functional theory (DFT) were carried out using the Vienna Ab Initio Simulation Package (VASP) [33]. The electronic exchange–correlation interactions were treated with the Heyd–Scuseria–Ernzerhof (HSE) hybrid functional [34,35], and the projector augmented-wave (PAW) [36] method was used to model electron-ion interactions. A plane-wave cutoff energy of 400 eV was adopted for all calculations. A 4 × 4 supercell of graphene was utilized to model mono-vacancy, di-vacancy, and tri-vacancy defects. This supercell size has been widely adopted in numerous studies [37,38]. Meanwhile, due to finite-size effects, interactions exist between defect images under periodic boundary conditions, which may lead to weak coupling of electronic wavefunctions and the resulting slight band dispersion. Such effects are generally difficult to completely avoid in defect-containing systems. To avoid interlayer interactions under periodic boundary conditions, we set a vacuum layer of 15 Å in the unit cell. To ensure computational accuracy, a 9 × 9 × 1 k-point mesh was generated using the Gamma-centered method for sampling the Brillouin zone. The convergence thresholds for electronic energy and ionic forces were set to 10^−5^ eV and 0.01 eV/Å, respectively.

### 2.2. Modeling of Defects

**Figure 1:**
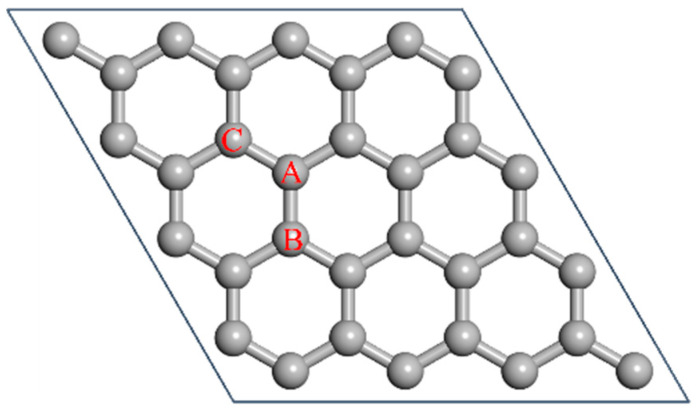
Schematic illustrations of carbon vacancy defects in graphene. Mono-vacancy, Di-vacancy, and Tri-vacancy defects were constructed by removing atoms at sites A, A-B, and A-B-C, respectively, based on a 4 × 4 supercell model containing 32 carbon atoms.

To quantify the influence of different vacancy defects on the piezoresistive properties of graphene, it is necessary to construct defective graphene models with specific vacancy types. In this study, we systematically constructed mono-vacancy, di-vacancy, and tri-vacancy defect models by progressively removing specific carbon atoms from the lattice. As illustrated in Figure 1, the mono-vacancy defect was generated through removal of the atom located at position A, while the di-vacancy defect was created by sequential removal of atoms at positions A and B, followed by additional removal of the atom at position C to form the tri-vacancy defect, resulting in three distinct vacancy configurations. Through this approach, a series of vacancy-defective graphene models were established, thereby laying the foundation for subsequent investigations of electrical characteristics and piezoresistive properties.

## 3. Results and Discussion

### 3.1. Structural Properties

**Figure 2:**
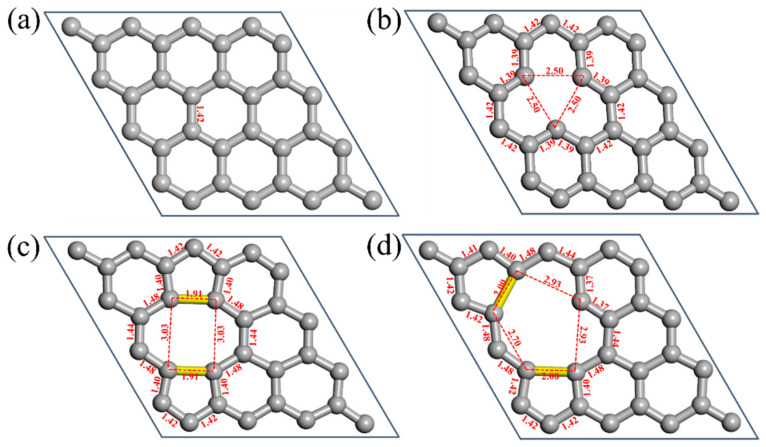
Relaxed structures of pristine and vacancy-defective graphene: (**a**) pristine, (**b**) mono-vacancy, (**c**) di-vacancy, and (**d**) tri-vacancy.

To investigate the influence of different types of vacancy defects on the graphene lattice structure, structural relaxation was performed on both pristine and defective graphene models. Figure 2a shows the relaxed pristine graphene, which has a C–C bond length of 1.42 Å. This value is in excellent agreement with established theoretical and experimental data, consistent with a typical sp^2^-hybridized carbon network [39]. As shown in Figure 2b, in the mono-vacancy system, the bond length between the two nearest carbon atoms adjacent to the vacancy shortens to 1.39 Å, which indicates an increase in bond strength. Concurrently, the atomic distance within the surrounding triangular configuration increases from 2.46 Å to 2.50 Å. This relaxation is driven by the Jahn–Teller effect, which lowers the local symmetry to saturate the partial dangling bonds, thereby reducing the total energy of the system [40,41]. As can be seen from Figure 2c, the di-vacancy defect results in bond lengths between the surrounding carbon atoms distributed between 1.40 and 1.48 Å, while the atomic spacings in the rectangular configuration around the vacancy become 1.91 Å and 3.03 Å, respectively, suggesting the possible formation of a 5-8-5 configuration here. As illustrated in Figure 2d, the tri-vacancy defect induces a more complex lattice distortion. This is manifested by a significantly broader distribution of bond lengths (1.37–1.48 Å) around the carbon atoms and by atomic spacings of 2.00 Å, 2.70 Å, and 2.93 Å in the pentagonal configuration, which points to the possible formation of a 5-10-5 structure. These results indicate that as the defect in graphene evolves from a single vacancy to multiple vacancies, the degree of lattice distortion intensifies significantly. Once the number of vacancy atoms exceeds one, the structure reconstructs to form non-hexagonal rings, which further disrupts the structural symmetry.

Subsequently, we calculated the formation energies of the various vacancy defect configurations. The calculation formula is as follows:

(1) $$E_{f}=E_{defect}-E_{pristine}+n\times \mu_{C}$$

where *E_defect_* and *E_pristine_* are the total energies of the defective and pristine graphene supercells, respectively, *n* is the number of carbon atoms removed, and *μ_C_* is the chemical potential of carbon, taken as the energy per atom in pristine graphene.

The calculation results indicate that the formation energies of the mono-vacancy, di-vacancy, and tri-vacancy defects are 7.9 eV, 8.2 eV, and 12.0 eV, respectively. This trend demonstrates that the energy required to form vacancy defects in graphene increases with the number of missing atoms. It is noteworthy that the average formation energy per vacancy atom for the di-vacancy and tri-vacancy defects is significantly reduced to 4.1 eV and 4.0 eV, respectively, which is much lower than the value of 7.9 eV for the mono-vacancy. This is primarily attributed to their reconstruction process, which forms new saturated chemical bonds, thereby effectively eliminating the high-energy dangling bonds. This bond reconstruction largely compensates for the energy loss caused by atom removal, making the overall structure more stable (Table 1).

### 3.2. Electronic Properties

**Figure 3:**
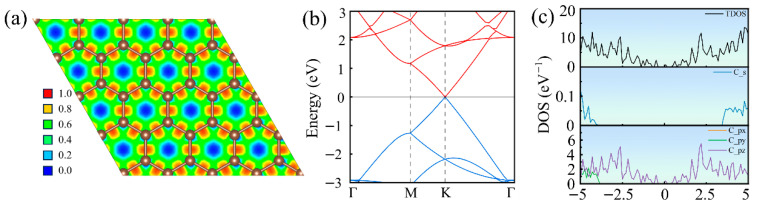
Electronic structure of pristine graphene: (**a**) Electron localization function, (**b**) electronic band structure, and (**c**) density of states.

To validate the reliability of our computational model, we first calculated the electronic structure of pristine graphene. As shown in Figure 3a, the electrons are uniformly distributed across the graphene plane without significant localized regions, indicating that the π-electrons are highly delocalized and exhibit metallic high conductivity. Figure 3b demonstrates that the band structure calculated along the high-symmetry path Γ–M–K–Γ in the Brillouin zone features the valence band maximum and the conduction band minimum intersecting precisely at the K point, forming a characteristic Dirac cone. This is consistent with the well-known zero-gap semimetallic nature [42,43] of pristine graphene, whose linear energy-momentum dispersion governs Dirac fermion behavior and high carrier mobility. Based on the calculated density of states, we further analyzed the contributions of individual electron orbitals to the band structure. As indicated in Figure 3c, the electronic states near the Fermi level are predominantly contributed by the pz orbitals of carbon atoms, demonstrating that the formation of the Dirac cone and the low-energy excitation processes primarily originate from the role of delocalized π-electrons. Meanwhile, the contributions of the px and py orbitals near the Fermi level are significantly smaller and completely degenerate, consistent with the robust σ-bond framework formed through sp^2^ hybridization, while the pz orbitals independently constitute the conductive π-system perpendicular to the planar direction. These results confirm the electronic structural characteristics of pristine graphene and provide a comparative basis for subsequent defect studies.

**Figure 4:**
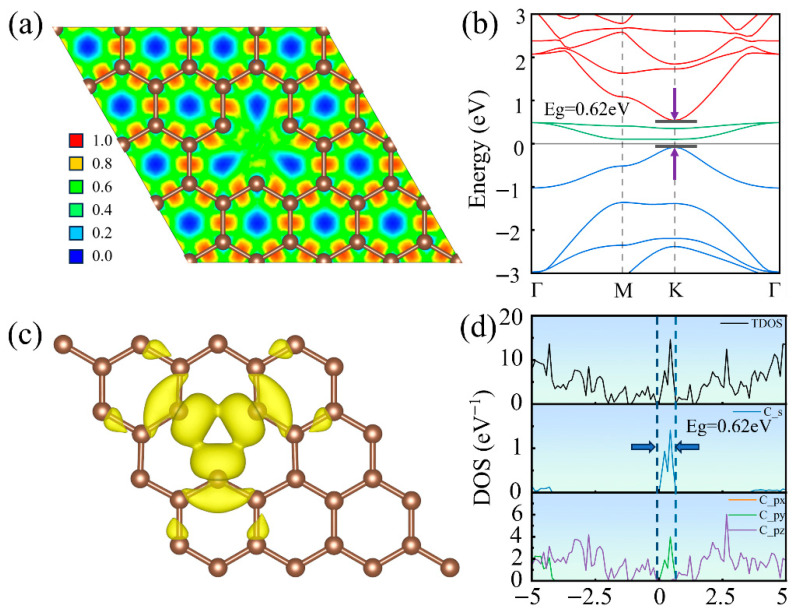
Electronic structure of mono-vacancy defective graphene: (**a**) Electron localization function, (**b**) electronic band structure, (**c**) charge distribution of defect states, and (**d**) density of states.

Figure 4a presents the electron localization function map, which shows reduced electron density around the missing carbon atom because the removal of the atom disrupts the surrounding σ and π bonding network, preventing electrons from forming a delocalized conjugated system. This reduction in local electron density leads to repulsive interactions between adjacent carbon atoms, resulting in an increase in their atomic spacing. As described in Figure 4b, the calculated band structure of the mono-vacancy defective graphene reveals that the breakdown of the periodic potential removes the degeneracy of the Dirac cone, which opens a bandgap of approximately 0.62 eV at the K point. Simultaneously, two relatively flat and localized defect-induced energy levels appear near the Fermi level. The corresponding charge distribution of the defect level is shown in Figure 4c, where the charge is predominantly localized around the defect atoms, thus confirming that the emergence of these defect levels is indeed caused by the introduction of the vacancy. As described in Figure 4d, the sharp density of states peaks near the Fermi level primarily originate from the s, px, and py orbitals, indicating that the mono-vacancy defect disrupts the sp^2^-hybridized network, leading to rehybridization of the s and p orbitals and resulting in defect states within the original π-π* energy gap. Moreover, since the px and py orbitals remain highly degenerate, the defect does not alter the overall in-plane symmetry of the graphene lattice.

**Figure 5:**
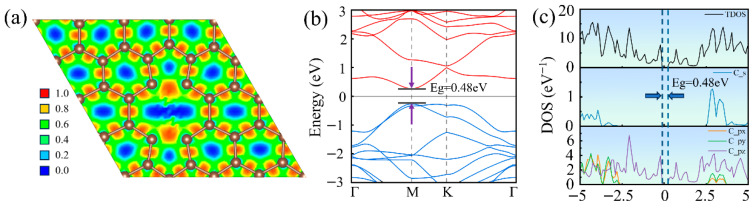
Electronic structure of di-vacancy defective graphene: (**a**) Electron localization function, (**b**) electronic band structure, and (**c**) density of states.

As shown in Figure 5a, the electron charge is highly localized between carbon atoms separated by 1.91 Å. This suggests the likely formation of a covalent bond between them, thereby providing theoretical evidence for the 5-8-5 configuration presented in Figure 2c. It can be observed from Figure 5b that the Dirac cone characteristics have completely disappeared in the calculated band structure of di-vacancy defective graphene. Due to the disruption of the periodic potential, the formation of non-hexagonal rings perturbs the delocalized π-electron system, and the defect-induced local potential causes a redistribution of the electronic eigenstates, leading the system to undergo band hybridization and renormalization. This process shifts the original Dirac point from the K point to the M point in the Brillouin zone; additionally, it results in the opening of a direct bandgap of 0.48 eV near the Fermi level. As shown in Figure 5c, the electronic state distribution reveals that, compared to pristine graphene, the pz orbitals undergo significant distortion near the Fermi level, exhibiting distinct peak-valley separation and an inability to form a continuous π-bond network. Meanwhile, the degeneracy between the px and py orbitals is also partially broken. Unlike the case of the mono-vacancy, these features indicate that the di-vacancy induces more substantial in-plane reconstruction, which has perturbed the symmetry of the sp^2^-hybridized network to a certain extent.

**Figure 6:**
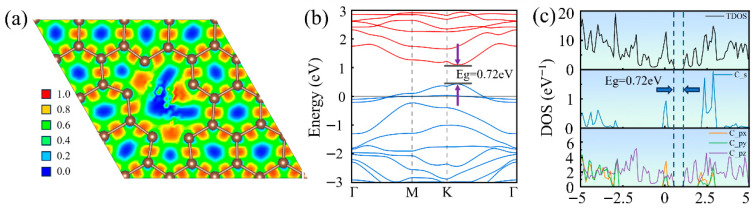
Electronic structure of triple-vacancy defective graphene: (**a**) Electron localization function, (**b**) electronic band structure, and (**c**) density of states.

Furthermore, we calculated the electron localization function, band structure, and density of states for the tri-vacancy defective graphene. As shown in Figure 6a, the electron localization function reveals highly localized electron charge between carbon atoms with a separation of 2.00 Å, suggesting the likely formation of a covalent bond between them, which specifically corresponds to the 5-10-5 configuration in Figure 2d. Figure 6b demonstrates that the triple vacancy defect fundamentally reconstructs the electronic band structure of graphene. The Dirac points of the new bands are no longer restricted to high-symmetry points; instead, they shift along the K–Γ path between the K and Γ points to attain the lowest energy configuration. The linear dispersion of the Dirac cone is further disrupted, resulting in the opening of a direct bandgap of 0.72 eV. Meanwhile, the Fermi level shifts below the valence band maximum, indicating that the system exhibits p-type conduction with holes as the primary charge carriers. Figure 6c shows that the electronic states near the Fermi level are co-contributed by both the s and p orbitals of carbon atoms. Their density of states distribution exhibits broadening and multi-peak characteristics, consistent with the diversity of defect configurations and the strong localization of electronic states. Meanwhile, the degeneracy between the px and py orbitals is further broken, accompanied by distortion and reconstruction of the in-plane structure.

**Figure 7:**
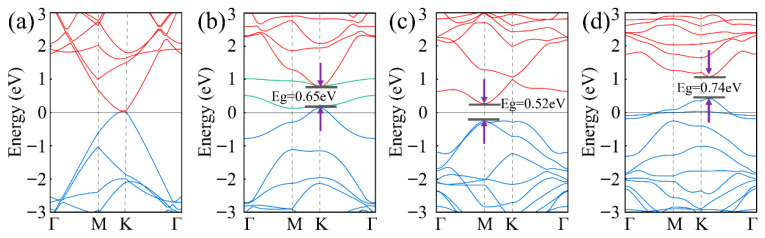
Electronic band structures of different models under 0.3% strain: (**a**) Pristine graphene, (**b**) mono-vacancy, (**c**) di-vacancy, and (**d**) tri-vacancy.

To investigate the role of vacancy defects in modulating the piezoresistive effect of graphene, a strain of 0.3% was applied in this study. This strain value was selected based on the operating conditions of the beam-type pressure sensors fabricated by our research team, which have a maximum strain of 0.3%. Within the regime of small deformations, strain modulates the hopping integral between adjacent carbon atoms by altering the carbon–carbon bond lengths. Since a strain of 0.3% is well below the linear elastic limit of graphene, the variation in the hopping integral exhibits a linear dependence on the strain magnitude, thereby ensuring qualitative consistency in band symmetry, density of states distribution, and transport behavior. Therefore, the physical behavior observed at 0.3% strain may, to some extent, be applicable to the entire small-strain regime. The influence of this strain on the electronic band structure of graphene was calculated. The results demonstrate that although no bandgap opens in pristine graphene under strain, the linear dispersion relation of the Dirac cone near the K-point is slightly weakened. For defective systems, strain further modulates their intrinsic electronic properties: the band gap of single-vacancy-defect graphene increases from 0.62 eV to 0.65 eV, while inducing the movement of defect energy levels towards the band edges, that of the di- and tri-vacancy defective graphene rises from 0.48 eV to 0.52 eV and from 0.72 eV to 0.74 eV, respectively. This is because the applied strain alters the bond lengths and bond angles of carbon atoms near the defects, inducing local lattice relaxation, which in turn leads to a redistribution of electronic states and a reconfiguration of the band structure, resulting in a slight modification of the overall electronic bands (Figure 7).

### 3.3. Piezoresistive Effect

The piezoresistive effect refers to the physical phenomenon where the electrical resistance of a material changes under mechanical strain, serving as the core mechanism for electromechanical signal conversion in pressure sensors [44]. The Gauge Factor (*GF*) is a key parameter for evaluating piezoresistive performance, defined by the formula [45]:

(2) $$GF=\frac{∆R/R}{\varepsilon }$$

where *R* is the initial resistance, *ε* denotes the strain, and Δ*R* represents the change in resistance.

Lei et al. further derived this formula, with the result shown below [46]. The detailed derivation process is provided in the Appendix A:

(3) $$GF=1+\nu -2\frac{\Delta V_{f}/V_{f}}{\varepsilon }$$

It should be noted that the relationship between resistivity and Fermi velocity adopted in the Appendix A is based on a simplified semiclassical transport model. Within this framework, the electrical conductivity is expressed as follows: σ=neμ, where the carrier mobility is mainly governed by the band dispersion. By further assuming that the carrier concentration and relaxation time remain approximately constant under small strain, the resistivity can be approximated to be inversely proportional to the square of the Fermi velocity.

Where ν is the Poisson’s ratio of the material, *V_f_* is the Fermi velocity, and Δ*V_f_* denotes the change in Fermi velocity induced by strain. The Fermi velocity is calculated by the following formula [47]:

(4) $$V_{f}=\frac{1}{\hbar }\frac{dE}{dK}$$

in which ℏ is the reduced Planck’s constant, and *dE*/*dK* represents the derivative of energy with respect to momentum.

The calculated Fermi velocities are summarized in Table 2. Analysis indicates that under strain-free conditions, the introduction of vacancy defects leads to a monotonically decreasing trend in Fermi velocity: from 1.047 × 10^6^ m/s in pristine graphene to 0.847 × 10^6^ m/s for mono-vacancy, 0.700 × 10^6^ m/s for di-vacancy, and 0.440 × 10^6^ m/s for tri-vacancy, respectively. This trend primarily stems from the disruption of the linear dispersion relation of the Dirac cone induced by the defects, along with the resulting band flattening and electron localization effects. These changes are equivalent to an increase in the electron effective mass, ultimately leading to a reduction in Fermi velocity and a decrease in carrier mobility. Furthermore, upon applying a small strain of 0.3%, the Fermi velocities of all systems exhibit a further decrease, indicating that strain introduces an additional perturbative effect on the defect-modified electronic structure.

Based on the aforementioned changes in Fermi velocity, we calculated the gauge factors for the different models. As shown in Table 3, the gauge factors for pristine graphene, mono-vacancy, di-vacancy, and tri-vacancy defective graphene are 2.37, 8.28, 4.91, and 13.22, respectively. Notably, the tri-vacancy defect yields the most significant enhancement, increasing the gauge factor by a factor of 5.58 compared with pristine graphene, which is far higher than the value reported by Yokaribas et al. for liquid-phase exfoliated graphene nanoplatelets (G = 5.6) [48]. The underlying physical mechanism is primarily that vacancy defects disrupt the local σ/π bonding network, inducing electron localization and the formation of defect states. This localization perturbs the linear dispersion of the Dirac cone and reduces the Fermi velocity, which restricts carrier mobility and makes the carriers more sensitive to applied strain, thereby significantly enhancing the piezoresistive response. These results clearly demonstrate that the introduction of vacancy defects can significantly enhance the piezoresistive sensitivity of graphene, and the extent of this enhancement depends on the specific configuration of the defects.

## 4. Conclusions

This study systematically investigates the modulation mechanisms of mono-vacancy, di-vacancy, and tri-vacancy defects on the electrical and piezoresistive properties of graphene, based on first-principles calculations. It is confirmed that di- and tri-vacancy defects reconstruct into 5-8-5 and 5-10-5 configurations, respectively, during structural relaxation. Vacancy defects can significantly alter the electronic structure of graphene by opening bandgaps, introducing localized defect levels, and shifting the Dirac points. Strain can effectively modulate the electrical properties of defective graphene, causing an increase in the band gap and movement of the defect energy levels in single-vacancy graphene toward the band edges. More importantly, the enhancement of the piezoresistive performance induced by vacancy defects displays a non-monotonic, configuration-dependent behavior. The gauge factors for mono-, di-, and tri-vacancy defects are 8.28, 4.91, and 13.22, respectively, with the tri-vacancy defect producing the most significant enhancement, enhancing the gauge factor by a factor of 5.58. These findings confirm the feasibility of designing high-performance graphene pressure sensors by controlling defects, laying a solid theoretical foundation for future development in this field.

## Figures and Tables

**Table 1:** Formation energy of vacancy defect for graphene.

Vacancy Model	MV	DV	TV
Formation energy (eV)	7.9	8.2	12.0
Formation energy (eV/C atom vacancy)	7.9	4.1	4.0

**Table 2:** Fermi velocity of different models before and after applying strain.

Vacancy Model		Graphene	MV	DV	TV
Fermi velocity (10^6^ m/s)	Strain-free	1.047	0.847	0.700	0.440
	0.3% strain	1.045	0.838	0.696	0.432

**Table 3:** Gauge factors of graphene under different models.

Vacancy Model	Gra	MV	DV	TV
Gauge Factor	2.37	8.28	4.91	13.22

## Data Availability

The data that support the findings of this study are available from the corresponding author upon reasonable request.

## Appendix A. Derivation Process of GF

The Gauge Factor is one of the key parameters for evaluating the performance of a piezoresistive pressure sensor and is defined as:

(A1) $$GF=\frac{∆R/R}{\varepsilon }$$

According to

(A2) $$R=\rho \frac{L}{S}$$

where *R*, *ρ*, *L*, and *S* represent the resistance, resistivity, length, and cross-sectional area of the material, respectively, the following relationship can be deduced:

(A3) $$\frac{∆R}{R}=\frac{∆L}{L}-\frac{∆S}{S}+\frac{∆\rho }{\rho }$$

Since the thickness of graphene does not change under uniaxial strain, we obtain:

(A4) $$\frac{∆S}{S}=\frac{h∆w}{h\;w}=\frac{∆w}{w}$$

Owing to the inverse proportionality between the resistivity and the square of the Fermi velocity, the following expression can be derived:

(A5) $$\rho =\frac{1}{n_{e}\;e\;\mu_{e}}=\frac{c}{{V_{f}}^{2}}$$

where *n_e_*, *e*, *μ_e_* and *c* represent the carrier concentration, carrier mobility, electron charge, and a constant, respectively.

Therefore, when the applied strain is small and Δ*V_f_* is infinitely small relative to *V_f_*, the following expression can be obtained:

(A6) $$\frac{∆\rho }{\rho }=-2\frac{{∆V}_{f}}{V_{f}}$$

Substituting Equations (A4) and (A6) into Equation (A3), we obtain:

(A7) $$\frac{∆R}{R}=\frac{∆l}{l}-\frac{∆w}{w}-2\frac{{∆V}_{f}}{V_{f}}$$

From the fundamental definitions of strain and Poisson’s ratio, the following relationship is obtained:

(A8) $$\varepsilon =\frac{∆l}{l}$$

(A9) $$\frac{∆w}{w}=-\nu \frac{∆l}{l}$$

where ε is the strain, and ν is the Poisson’s ratio. Equation (A7) can be expressed as follows:

(A10) $$\frac{∆R}{R}=\varepsilon +\nu \varepsilon -2\frac{{∆V}_{f}}{V_{f}}$$

Substituting Equation (A10) into the GF expression, we finally arrive at:

(A11) $$GF=1+\nu -2\frac{\Delta V_{f}/V_{f}}{\varepsilon }$$
